# Endothelial *Erg* Regulates Expression of Pulmonary Lymphatic Junctional and Inflammation Genes in Mouse Lungs Impacting Lymphatic Transport

**DOI:** 10.21203/rs.3.rs-3808970/v1

**Published:** 2024-01-24

**Authors:** Adri Chakraborty, Alex Kim, Salam AlAbdullatif, Joshua D. Campbell, Yuriy O. Alekseyev, Ulas Kaplan, Vrinda Dambal, Giovanni Ligresti, Maria Trojanowska

**Affiliations:** 1 Arthritis & Autoimmune Diseases Research Centre, Department of Medicine, Boston University Chobanian & Avedisian School of Medicine, Boston, MA, USA; 2 Division of Computational Biomedicine, Department of Medicine, Boston University Chobanian & Avedisian School of Medicine, Boston, MA, USA; 3 Department of Pathology and Laboratory Medicine, Boston University Chobanian & Avedisian School of Medicine, Boston, MA, USA

## Abstract

The ETS transcription factor ERG is a master regulator of endothelial gene specificity and highly enriched in the capillary, vein, and arterial endothelial cells. ERG expression is critical for endothelial barrier function, permeability, and vascular inflammation. A dysfunctional vascular endothelial ERG has been shown to impair lung capillary homeostasis, contributing to pulmonary fibrosis as previously observed in IPF lungs. Our preliminary observations indicate that lymphatic endothelial cells (LEC) in the human IPF lung also lack ERG. To understand the role of ERG in pulmonary LECs, we developed LEC-specific inducible *Erg*-CKO and *Erg*-GFP-CKO conditional knockout (CKO) mice under *Prox1* promoter. Whole lung microarray analysis, flow cytometry, and qPCR confirmed an inflammatory and pro-lymphvasculogenic predisposition in *Erg*-CKO lung. FITC-Dextran tracing analysis showed an increased pulmonary interstitial lymphatic fluid transport from the lung to the axial lymph node. Single-cell transcriptomics confirmed that genes associated with cell junction integrity were downregulated in *Erg-*CKO pre-collector and collector LECs. Integrating Single-cell transcriptomics and CellChatDB helped identify LEC specific communication pathways contributing to pulmonary inflammation, trans-endothelial migration, inflammation, and Endo-MT in *Erg*-CKO lung. Our findings suggest that downregulation of lymphatic *Erg* crucially affects LEC function, LEC permeability, pulmonary LEC communication pathways and lymphatic transcriptomics.

## Introduction:

Lymphatics in the lungs comprise a complex network of vascular structures that contribute to immune modulation, tissue fluid clearance, and macromolecule transport ^1,2^. Fibrosis of lungs is attributed to pathologic changes and lesions in the parenchyma and is accompanied by vascular and lymphatic vessel remodeling. Homeostatic dysregulation of angiogenic and lymphangiogenic interstitial fluid balance transport accentuates excessive trafficking of antigen presenting cells (APCs), cytokines and promotes tissue fibrosis^3,4^. Lymphangiogeneis has been previously considered a negative prognostic marker and an ongoing indicator of pulmonary fibrosis^5^, however, protective role of lymphatics against lung injury and fibrosis exits ^6,7^. A high number of lymphatic vessels have been observed in fibrotic NSIP and UIP, although lymphatic remodeling/localization, but not the degree of lymphangiogenesis, is considered as a reliable deterministic factor of fibrosis severity^5,8^. Fibrotic foci in lung is in-fact devoid of lymphatic structures ^9,10^ and were predominantly found localized in the alveolar lesions ^11^. More-specifically, tissue fibrosis impairs lymphatic regeneration^12^. Dysfunctional lymphatic drainage in fibrotic lung tissues results in imbalance in interstitial fluid and immune cell homeostasis and thus promotes fibrosis.

Several studies have reported lymphatic remodeling in patients with idiopathic pulmonary fibrosis (IPF). Histopathological studies of lymphatics in IPF demonstrated alveolar lymphangiogenesis and increased vessel diameter correlated with disease severity ^4,9,11^. Furthermore, a stereological analysis of both lymphatic and parenchymal structures found strong correlations between lymphatic length and fibrotic collagen density, consistent with lymphangiogenesis during fibrosis ^13^. These studies suggest that lymphatic remodeling plays a critical role in lung fibrosis in IPF. Lymphatic dysfunction could therefore be a predictive indicator of fibrotic pulmonary stages and progression.

Despite the differences in the pathogenesis of lung fibrosis between humans and mice, mouse models have been useful in illustrating the role of lymphatics in the disease’s pathogenesis. Baluk et al ^6^ recently illustrated lymphatic expansion and remodeling in a bleomycin model of lung fibrosis that contributes to the resolution of fibrosis. Furthermore, transgenic overexpression of VEGFC reduced inflammation and accelerated recovery after bleomycin treatment, suggesting a protective role of the lymphatic system in lung fibrosis by reducing lymph stasis and accelerating clearance of fluid and cells.

The ETS transcription factor ERG is a major regulator of endothelial homeostasis, extracellular matrix remodeling, and suppressor of pro-inflammatory genes ^6,14,15^. ERG expression is critical for vascular integrity, endothelial permeability, and blood endothelial transcriptional regulation ^16^. Loss of pulmonary vascular endothelial ERG signaling impacts immune and endothelial functions resulting in persistent fibrosis in the lungs ^14,17,18^. ERG is a master regulator of endothelial gene specificity and regulates the transcription of several endothelial-specific genes (*von Willebrand factor, Icam2, Ve-cadherin, Aplnr,* etc.). ERG expression is critical for endothelial barrier function, permeability, and vascular inflammation ^16,19^. In pulmonary arterial hypertension (PAH), which involves vascular inflammation and pathological angiogenesis, ERG expression is significantly reduced in pulmonary endothelial cells ^20^. ERG also regulates immune tolerance T-helper responses in allergic pulmonary responses, B cell NFKB1 activation ^14,18^, and pulmonary fibroblast activation during aging ^17^. ERG is highly expressed in lymphatic endothelial cells (LEC) although its role in lymphatic function is largely unknown ^21^.

Given the critical function of ERG in vascular endothelial cells, the goal of this study was to gain insights into the role of ERG in lymphatic cells, focusing on pulmonary lymphatics. We report that pulmonary lymphatic ERG dysfunction increases lymphatic permeability, upregulates expression of genes associated with inflammation, and downregulates LEC junctional integrity genes. Further, we show that inflammation concurs an increased pulmonary interstitial fluid transport. Mice *Erg*-CKO LECs upregulate ER stress and oxidative phosphorylation genes and pathways consistent with stressed IPF LECs. In vitro ERG regulates the expression of lymphatic identity gene (*Prox1*) and promotes pro-inflammatory genes.

## Results:

### Human IPF lymphatic vessels lose *Erg* expression.

**Figure 1:**
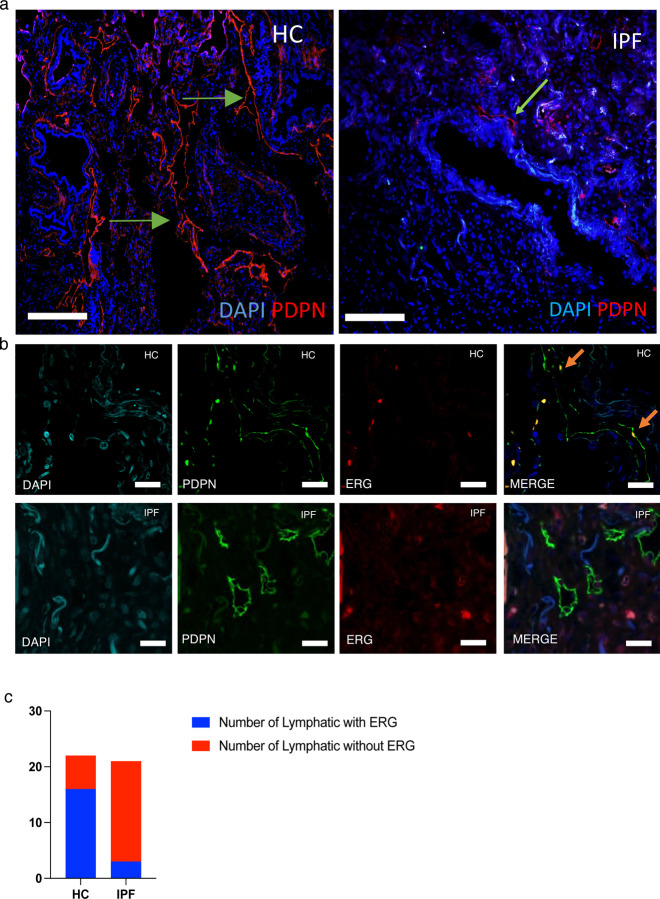
ERG expression in human IPF lungs. **a,** Immunofluorescence of PDPN+ lymphatics in healthy control (n=3) and IPF human lungs (n=4). **b,** ERG expression in PDPN+ lymphatic vessels characterized through immunofluorescence in HC and IPF lungs (n=6 vs 6). **c,** Representative counts of lymphatic vessels with nuclear ERG expression in IPF lungs suggesting a loss of ERG expression compared to IPF lungs.

Fibrotic lung diseases often display abnormal vascular remodeling and changes in lymphatic numbers and morphology in the lung parenchyma. These physiological changes may be accompanied by dysfunctional immune response and cell communication, as observed in IPF lungs ^4,9,22^. Our analysis of lymphatic vessels in 4 IPF and 3 healthy control lungs showed small disjointed lymphatic vessels within the alveolar space of human IPF lung (Fig 1a) corroborating previous reports ^9^. Previous studies have also shown that ERG expression is critical for vascular integrity, although, its suggested role in LEC junctional integrity is merely speculative ^16^. In the absence of endothelial ERG expression, signaling associated with lineage specificity, sheer stress, cell migration and inflammation is dysregulated in BECs ^23,24^, however the molecular function of ERG in LECs is unknown. Given the well-known role of ERG in angiogenesis and endothelial lineage specific gene expression ^25^, we next assessed the expression of ERG in the lymphatic vessels in IPF lungs. Interestingly, compared to healthy lung lymphatics, IPF lymphatic structures had a 10-fold decrease in nuclear ERG expression (Fig. 1b, c). To investigate the role of LEC-ERG, we generated a LEC specific conditional knockout mice using tamoxifen inducible Cre under the *Prox1* promoter (*Prox1-Cre/*^*ERT2/*^*Erg*^*fl/fl*^,) (*Erg*-CKO mice). WT and *Erg*^*fl/fl*^ */Prox1-Cre/*^*ERT2*^ mice were also bred to the *tdTomato-Egfp* (mT/mG) (Jackson lab) reporter mice to enable GFP expression in the LECs (WT/ *Erg*-CKO-GFP mice).

### *Erg* downregulation in pulmonary LECs affects lymphatic remodeling, gene expression, and drainage function.

**Figure 2:**
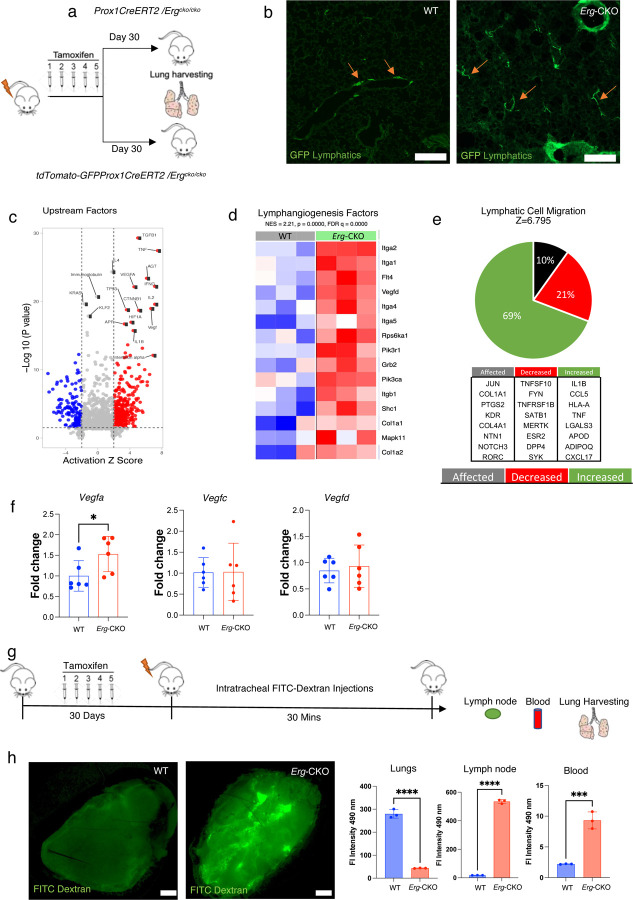
Erg downregulation in pulmonary LECs affects lymphatic morphology, function, and gene expression. **a,** Conditional *Erg*^fl/fl^ /Prox1-Cre/^ERT (^Erg-CKO) on a C57/BL6 background were generated by breeding of Prox1-CreER(T)-mice with Erg^fl/fl^ mice. **b,** Visualization of GFP expressing lymphatic vessels in WT-GFP and *Erg*^fl/fl^ /Prox1-Cre/^ERT2^-GFP lung parenchyma (n = 3 WT vs 3 *Erg-*CKO). **c,** RNA-Seq analysis: Volcano plot: the x-axis represents activation Z score, the y -axis represents the corrected significance level after base log10 conversion. Red dots in the figure indicate the factors activated in *Erg*-CKO mice lungs (NES=2.21). **d,** RNA-Seq differential gene expression analysis identifies collective upregulation of lymphangiogenic genes in *Erg*-CKO lungs (NES=2.21). **e,** Ingenuity pathway metadata analysis displaying gene aggregation contributing to lymphatic cell migration in *Erg-*CKO lungs −log p-value > 2; z-score > 5; n = 3 WT vs 3 *Erg-*CKO). **f,** Relative RNA expression of key lymphangiogenic factors (VEGF-A, VEGF-C, VEGF-D) from WT and *Erg*-CKO total lungs (n = 6 WT vs 6 *Erg-*CKO). **g,** Intratracheal administration of 250 kDa FITC-Dextran in WT and *Erg*-CKO lungs, followed by analysis of dye retention in the lungs, axial lymph nodes and in systemic circulation. **g,** Representation of 250 kDa FITC-Dextran retention in the axial lymph nodes (green fluorescence) followed by fluorescent intensity analytics of whole tissue homogenate and blood measured at 488 nm (n = 3 WT vs 3 *Erg*-CKO; p < 0.05).

We sought to investigate if lymphatic ERG deficiency affects lung lymphatic function. 6-week-old WT-GFP and *Erg-*CKO-GFP mice were tamoxifen injected and thirty days later mice were sacrificed, and lungs were isolated to characterize pulmonary lymphatics (Fig. 2a). Immunohistology analysis of *Erg-*CKO-GFP lungs indicated *de novo* lymphatic remodeling and an increased distribution variance of lymphatic vessels throughout the lung interstitial space (Fig. 2b Supplementary Fig 1a, b, c). Few vessels expressed KI67, although no difference in KI67 expression levels in the WT and Erg-CKO lung lymphatics was observed (Supplementary Fig. 1d).

To determine changes in WT and *Erg-*CKO pulmonary gene signatures, whole lung microarray was performed followed by gene clustering using ingenuity pathway analysis (IPA) (Supplementary Fig. 1e). IPA clustering showed a consistent upregulation of genes associated with vasculogenesis, angiogenesis and LEC proliferation in *Erg-*CKO mice. Among the top fifteen upstream factors upregulated in lungs (represented as volcano plot: Z score >2) were genes involved in lymph-vasculogenesis (*Vegf, Vegfa*), inflammation (*Tnf, Ifng, Il2*), fibrogenesis (*Agt, Tgfb1*), and cell cycle regulation (*Ctnnb1*). Upregulation of these genes were suggestive of vascular remodeling and inflammatory predisposition of *Erg-*CKO mouse lungs (Fig. 2c). Gene Set Enrichment Analysis (GSEA) analysis further suggested a coordinated upregulation of lymphangiogenic genes in *Erg-*CKO mice (NES 2.21, FDR q=0) (Fig. 2d). Z-score cluster aggregation of collective upregulated and downregulated genes in *Erg-*CKO lungs indicated genes associated with LEC migration increase in *Erg*-CKO Lungs (Fig. 2e). We also observed upregulation of key lymphovascular remodeling genes, *Vegfa* and *Pdgfb* in *Erg-*CKO lungs using qPCR. No changes in *Vegfc* and *Vegfd* gene expression were observed. This suggested that *Vegfa* potentially drives lymphatic remodeling in *Erg-*CKO lungs (Fig. 2f).

Next, to determine if *Erg* downregulation in pulmonary lymphatics impacts fluid drainage, 2,000 kDa FITC-Dextran was injected intratracheally at a concentration of 10 mg/kg b.w ^26^. The quantity of dye retention in lungs v/s in circulation to axial lymph node and blood was monitored after 30 mins post injection (Fig 2g). We observed that the concentration of FITC-Dextran was 30 folds elevated in the axial lymph node, 4.5-fold in the systemic circulation in *Erg*-CKO mice, while there was a 5-fold reduction in the *Erg-*CKO lung interstitium (Fig. 2h). These findings suggest that downregulation of LEC-ERG expression facilitates increased fluid transport, which might be caused by an increased vessel permeability.

### *Erg* downregulation in LECs predisposes mice lungs to immune cell infiltration but not fibrosis.

**Figure 3:**
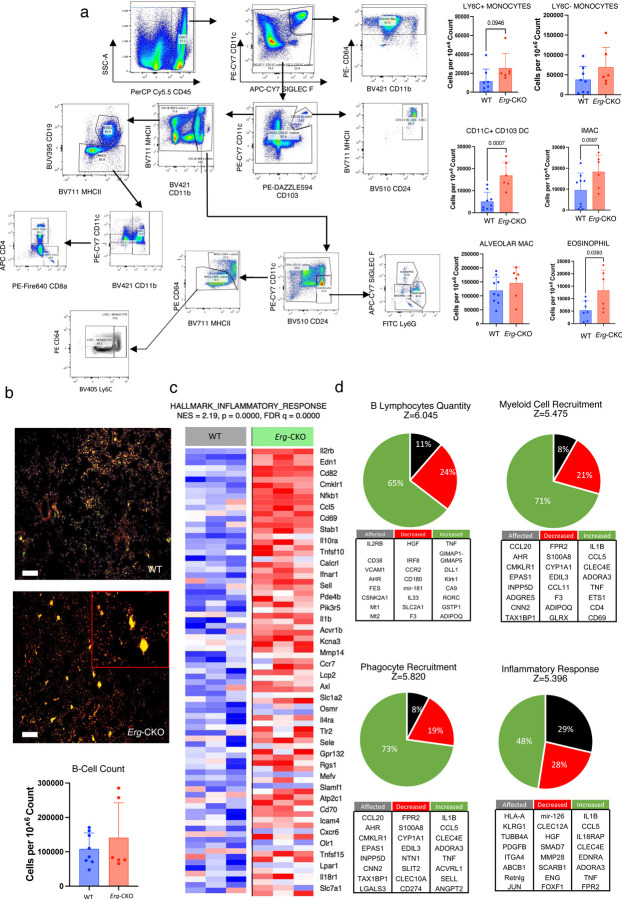
Erg downregulation in LECs predisposes mice lungs to inflammation. **a,** Flow-cytometric analysis of lymphoid and myeloid immune subsets in WT and *Erg*-CKO lungs (n = 8 WT vs 6 *Erg-*CKO.) **b,** Immunofluorescence of B220+ B-cell aggregates in *Erg*-CKO lungs (n = 8 WT vs 6 *Erg*-CKO). **c,** Whole lung microarray GSEA analysis displaying a collective upregulation of pulmonary inflammation genes in *Erg-*CKO lungs. **d,** Ingenuity pathway metadata analysis displaying gene aggregation contributing to upregulated pathways associated with lymphocyte quantity, myeloid cell recruitment, phagocyte recruitment and pro-inflammatory response in *Erg-*CKO lungs −log p-value > 2; z-score > 5; n = 3 WT vs 3 *Erg-*CKO).

Endothelial ERG expression is a critical inhibitory mediator of vascular inflammation and fibrosis^14,17,27^. We investigated if lymphatic ERG expression protects against vascular injury and inflammation. To examine if LEC ERG downregulation affects lung pathophysiology, we employed flow-cytometry and sequential gating to characterize immune cells from the lungs of 7 WT and 9 *Erg-*CKO mice first focusing on the myeloid subset ^28^ (Fig. 3a). Our flow-cytometric analysis of the *Erg-*CKO lungs confirmed an elevated presence of inflammatory LY6C+ monocytes ^29^, phagocytic LY6C- monocytes ^29,30^, pro-fibrotic and inflammatory interstitial macrophages ^31,32^, adaptive immune activator CD11C+ CD103+ dendritic cells ^33^ and eosinophils ^34^ (Fig 3a). Flow-cytometry did not indicate changes in B-cell numbers in *Erg-*CKO lungs (Fig. 3b). We did however observe large aggregates of B-cells clustered along the bronchioles of at least half of the *Erg-*CKO mice lungs (Fig. 3b). Whole lung microarray GSEA analysis showed a collective upregulation of genes involved in pulmonary inflammation in *Erg-*CKO lungs (Fig. 3c, Supplementary Fig. 2a). Ingenuity gene aggregation metadata analysis indicated an upregulation of genes associated with phagosome formation, leukocyte extravasation, B-lymphocyte quantity, pulmonary fibrosis/healing, and myeloid cell recruitment in *Erg-*CKO lungs (log p-value>2, z-score >5). (Supplementary Fig. 2b). We used Z-score aggregation to cluster the upregulated and downregulated genes and collectively determined signaling pathways activated in *Erg-*CKO lungs. We observed an increase in B-lymphocyte signaling, myeloid cell recruitment, phagocyte recruitment, pulmonary inflammation onset, and fibrogenesis (Fig. 3d, Supplementary Fig 2c). Additionally, qPCR confirmed upregulation of genes known to contribute towards systemic inflammation, and pulmonary function decline and fibrosis, including *Pai1*, *Mmp12, IfnγR2*, *Ccn2*, *Pdgfb,* and *Tgfb,* in *Erg*-CKO mice (Supplementary Fig. 2d). Although critical genes and pathways associated with fibrosis were upregulated in the lung, *Erg-*CKO mouse lungs did not display lung fibrosis suggesting anti-fibrotic effects of lymphatic fluid transport in *Erg*-CKO lungs. We also did not observe any changes in total hydroxyproline collagen content in *Erg-*CKO lungs (Supplementary Fig. 2e). Overall, our findings suggest that lungs with lymphatic endothelial ERG downregulation display increased immune cell infiltration but not sufficient to affect pulmonary fibrosis.

### Single Cell Sequencing supports LEC heterogeneity and changes in junctional integrity genes in *Erg-*CKO mice lungs.

**Figure 4:**
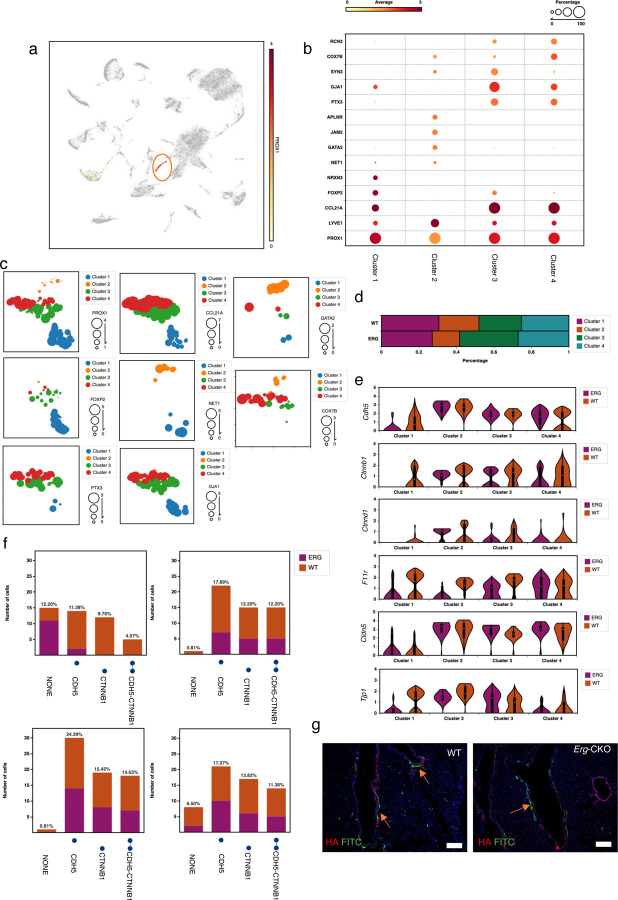
Single Cell Sequencing analysis (scRNAseq) analysis of whole lung cells isolated from *Erg-*CKO and WT mice (n = 2 WT vs 2 *Erg-*CKO). **a,** UMAP *Prox1* lymphatics clustered using Biotouring-BBrowserX cell type prediction. **b,** Canonical correlation analysis (CCA) based unsupervised Louvian clustering: gene signature-based clustering of LECs into four groups: cluster 1: pre-collectors, Cluster 2- Collectors, Cluster 3- iLECs Pre-capillary Lymphatics, Cluster 4- Capillary Lymphatics. **c,** UMAP distribution of gene expression in each of the four LEC subclusters. **d,** Cell count cluster distribution of LECs in WT and *Erg-*CKO lungs. **e,** Gene expression of tight and adherens junction genes in WT and *Erg-*CKO LEC subclusters. **f,** Gene correlation analysis showing overlapping gene distribution (*Cdh5, Ctnnb1*) in individual WT and *Erg-*CKO LECs. **g,** Immunohistochemistry of Hyaluronan staining in WT and *Erg-*CKO lymphatic structures (n = 3 WT vs 3 *Erg-*CKO).

To assess if ERG downregulation affects LECs transcriptomics, we performed scRNA-seq analysis of whole lungs isolated from *Erg-*CKO and WT mice. Using, Biotouring-BBrowserX unsupervised cell type prediction-marker based clustering with Seurat ^35^, we enriched *CD45-/Prox1+* lymphatic clusters (Fig. 4a). LECs were structurally segregated into four sub-clusters based on *Lyve-1* and *Ccl21a* expression pattern as follows: Cluster 1- Pre-collectors (*Prox1* high, *Ccl21a* low, *Flt4* high, *Nrxn3*+) Cluster 2- Collector Lymphatics (*Prox1* low, *Lyve1* high, *Jam-2+, Aplnr+*, *Nrp2* low, *Flt4* low), Cluster 3- interstitial LECs (iLECs) pre-capillary Lymphatics (*Prox1* high, *Vegfr2* high, *Ccl21a* high, *Gja1* high, *Cox7b* low, *Ptx3*+) and 4- capillary lymphatics (*Prox1* high, *Ccl21a* high, *Gja1* low, *Cox7b* high, *Ptx3+*) ^36–38^ (Fig 4b, Supplementary Fig 3a) and represented as UMAPs (Fig 4c). The capillary/pre-capillary and collector vessels were also validated using CCL21, PTX3 and LYVE1 staining respectively (Supplementary Fig 3b). Cell count distribution indicated variability across WT and *Erg*-CKO LEC clusters (Fig 4d). We also observed a significant downregulation of adherens junction genes, including VE-cadherin (*Cdh5)*, Beta-Catenin (*Ctnnb1*), Delta-Catenin (*Ctnnd1*) in the pre-collector *Erg-*CKO LECs (Fig 4e). Based on gene intersection analysis, all Erg-CKO pre-collector LECs that express *Cdh5* do not express *Ctnnb1.* The percentage of *Erg-*CKO pre-collector LECs expressing *Ctnnb1* was also zero. *Cdh5* expression levels in *Erg-*CKO pre-collector LECs was also 5-fold lower than WT LECs. In-addition, the number of *Erg-*CKO pre-collector LECs which loose *Cdh5* expression was 2-fold higher than WT LECs (Fig 4f). Gene correlation analysis indicated a positive correlation between *Cdh5* and *Cldn5* gene expression levels (Supplementary Fig 4a). Unlike previous studies, a majority of pre-collector and collector *Erg-*CKO LECs expressed low *Cdh5* gene or co-expressed low *Cdh5-Cldn5*, compared to WT LECs (Supplementary Fig 4b) ^39^. The percentage of LECs expressing *Zo-1 (Tjp1)* and *JamA (F11r)* was also lower in *Erg-*CKO pre-collector and collectors compared to WT LECs. In fact, both *Cdh5-F11r* and *Cdh5-Tjp1* co-expression was significantly reduced in Erg-CKO pre-collectors and collectors (Supplementary Fig 4c). Endo-MT marker gene expression was highly heterogeneous and no conclusion could be drawn regarding LECs undergoing Endo-MT in vivo. Interestingly, the expression of *Lyve1* was highest in the collector LECs, suggesting its role in possible metabolic degradation of hyaluronan and an increased uptake of fluids ^40^. Expression of *Lyve1* in collectors were validated through staining in Erg-Prox1-Gfp mice as previously mentioned in Supplementary Fig 3b. No significant changes in LEC adherens and tight junction genes expression levels were observed in the capillary and iLECs-pre-capillary lymphatics. Hyaluronan leaks in the tissue interstitium was not observed in *Erg-*CKO lungs suggesting no vascular and lymphatic permeability dysfunctions (Fig 4g).

### CellChat predicts key cell-cell communication networks in *Erg*-CKO lungs.

**Figure 5:**
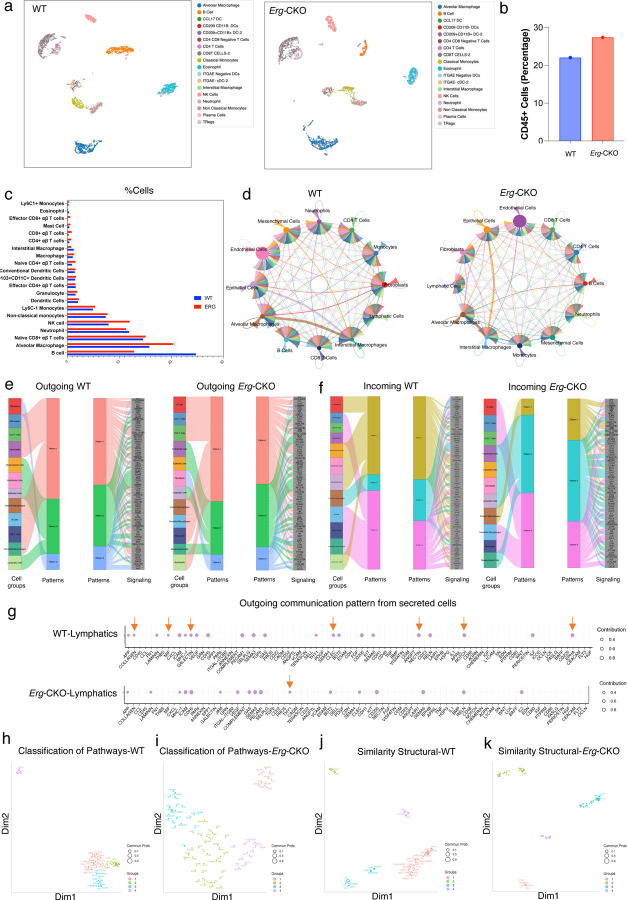
CellChat predicts key signaling molecule communication patterns in *Erg*-CKO lungs: Single-cell transcriptomics. **a,** UMAP projection of lymphoid and myeloid immune subsets in WT and *Erg*-CKO lungs (n = 2 WT vs 2 *Erg*-CKO). **b,** *Cd45+* cell counts in WT and *Erg*-CKO lungs (n = 2 WT vs 2 *Erg-*CKO). **c,** Percentage distribution of enriched lymphoid and myeloid immune subsets in WT and *Erg*-CKO lungs. **d,** CellChat ligand-receptor communication probability analysis between WT and *Erg-*CKO single cell populations suggesting the communication strength across cell types. **e,** CellChat river-plot analysis identifies outgoing signaling patterns from WT and *Erg-*CKO cell populations and the receptors involved, clustering them into groups based on strength of the signals. **f,** CellChat river-plot analysis identifies incoming signaling patterns to WT and *Erg-*CKO cell clusters and the receptors involved. **g,** Output communication receptors involved in upregulated pathways in WT and *Erg-*CKO LECs. **h,** CellChat NetSimilarity function identifying functional similarity of activated pathways in WT LECs. **i,** CellChat NetSimilarity function identifying functional similarity of activated pathways in *Erg-*CKO LECs. **j,** CellChat NetSimilarity structural similarity aggregation of WT LEC subclusters. **k,** CellChat NetSimilarity structural similarity aggregation of *Erg-*CKO LEC subclusters.

To gain further insight into how conditional *Erg* knockout affect the pulmonary LEC autocrine and paracrine communications pathways, we used CellChat quantitative analytics to understand ligand, receptors, and cofactor interactions between pulmonary lymphatics and other cell types. CellChat considers the structural composition of ligand-receptor interactions (such as multimeric ligand-receptor complexes, soluble agonists and antagonists, and dynamic interactions between signaling molecules at a system-wide level), identifies the key interactions that lead to cellular responses as well as stimulatory and inhibitory membrane-bound co-receptors, and compares it with KEGG pathway and peer-reviewed database. We started with dimension reduction and database clustering of WT and *Erg-*CKO single-cell datasets to identify flow-induced EC subpopulation heterogeneity based on CD45 populations. The following clustering parameters were used: immune cells (*Cd45+*) lymphatic endothelial, epithelial, and fibroblast cell population (*Cd45-*) (Supplementary Fig. 5a, b). The cell population enrichments are as follows: Alveolar Macrophages (*Ear2*), NK cells (*Gzma*), B cells (*Cd79b*), Plasma cells (*Mzb1*), CD4+ T cells (*Trbc2*), CD8+ T cells (*Trbc2, Cd8b1, Ly6c2*), Non-classical monocyte (*Ly6c2-,Plac8*), Interstitial macrophages (*C1qb*), Classical monocyte (*Ly6c2+, Plac8*), CD103+/CD11b-_DCs (*Itgae*), Ccl17+/CD103-/CD11b- DCs (*Ccl17*), CD209+/CD11b+ DCs (*Ccl17, Cd209a*), Tregs (*Cd3+, Cd4+, Foxp3+*), Neutrophils (*Ngp, Ly6c2*), Eosinophils (*Cxcr2*), Endothelial cells (*Cdh5, Pecam1*), Lymphatic endothelial cells (*Prox1*), Alveolar Fibroblasts (*Pdgfra, Slc7a10*), Adventitial Fibroblasts (*Pi16, Dcn*), Peribronchial Fibroblasts (*Fgf18, Hhip*), and Activated Fibroblasts (*Col1a1, Fn1*) (Supplementary Fig 5a, b). Dimension reduction with Uniform Manifold Approximation and Projection analysis identified transcriptomic changes in B cells, classical, non-classical monocytes, eosinophils, alveolar macrophages, and dendritic cell population between WT and *Erg-*CKO lungs (Fig 5a). While the overall percentage of *Cd45+* cells was 1.3-fold higher in the *Erg-*CKO mice lungs (Fig 5b), categorical distribution of the enriched cell subsets suggested an increase in alveolar macrophages, NK cells, and subsets of Naïve CD4+ αβ T cells and Effector CD8+ αβ T cells enriched in *Erg*-CKO lungs (Fig. 5c). B-cell enrichment was observed in WT mice lung, inconsistent with the flow cytometry data (Fig 5c).

CellChat was used next to infer and visualize cell-cell communication across *Erg-*CKO LECs and other cell identities. Approximately 2,021 validated molecular interactions were analyzed, including appx 60% of secreted autocrine/paracrine signaling interactions, 21% of extracellular matrix (ECM)-receptor interactions, and 19% of cell-cell contact interactions were analyzed and compared based on KEGG and molecular literature (Supplementary Fig 5c). We first calculated the aggregated cell-cell communication networks to determine the total interaction strength (weights) between any two cell groups using a circle plot. For broader understanding, the cell clusters were re-aggregated to look at the following populations: neutrophils, CD4+ T cells, CD8+ T cells, LECs, epithelial cells, endothelial cells, fibroblasts, B cells, alveolar macrophages, interstitial macrophages, monocytes, and mesenchymal cells. The communication probability between WT and *Erg-*CKO lung cell types is determined by the number of links with the thickness of the links determining communication strength (Fig 5d). Although the signaling output within the WT and *Erg-*CKO lung cell types were similar, the overall signaling strength was 10-fold lower in *Erg-*CKO mice compared to WT (Supplementary Fig 5d).

Our next step was to validate how the cells coordinate signaling pathways to communicate among themselves and other cell types. The signaling networks were identified using CellChat’s pattern recognition utility based on non-negative matrix factorization. This analysis produced a set of communication patterns that link cell groups with signaling pathways either in the context of incoming or outgoing signals. Three patterns of identified outgoing signals characterized in WT and *Erg-*CKO mice were the following: pathways associated with myeloid cells (pattern#1), endothelial, epithelial, lymphatic, mesenchymal cells (pattern#2), and fibroblast (pattern#3). The overall outgoing communication patterns of the secreted cells were dominated by pattern #1 (Fig. 5e, Supplementary Fig. 5e). The following ligands involved in outgoing communication were upregulated/downregulated in *Erg-*CKO populations: B cell-CD22, CD23, CD48 (upregulated), CD4+ T cells- CD226 (downregulated), CD8+ T cells- SPP1 (upregulated), Alveolar Macrophages- JAM, BST2 (upregulated), OCLN (downregulated), Interstitial macrophage- ICOS (downregulated), Monocytes- GALECTIN (downregulated), BST2 (upregulated) and Neutrophils- VISFASTIN (upregulated) (Supplementary Fig 5e). More specifically, output ligands associated signaling pathways upregulated in WT LECs included COLLAGEN, MIF, GALECTIN, CLEC, NECTIN, NOTCH, and EPHA. Outgoing signaling associated with THY1 receptor was upregulated in *Erg-*CKO LECs (Fig 5e, g). Combined incoming communication strength from endothelial, epithelial, fibroblast, lymphatics, and mesenchymal cells (pattern#1-WT, pattern#2- *Erg-*CKO) were the most dominant, followed by signaling from alveolar macrophage and myeloid cells (Fig 5f). A large proportion of incoming communication signaling involved receptors such as VEGF, PDGF, SEMA4, EPHA, and NECTIN (Fig 5f). More specifically, incoming communication signaling to *Erg-*CKO LECs were associated with an upregulation of lymphatic modulators and pro-inflammatory receptors such as PARs, HSPG, AGRN, and downregulation of OSM, ANGPT, and CD226 (Supplementary Fig 5f). Combining both incoming and outgoing communication pathways generated a comprehensive ligand-receptor interaction plots delineating LEC signaling function (Supplementary Fig 5g). The ligand-receptor interaction strength was five-fold lower in *Erg-*CKO lymphatics compared to the WT; however, THY1, SELL, VEGF, and SEMA6 receptor associated signaling was enriched. Similarly, NOTCH and GALECTIN receptor associated signaling was reduced in *Erg-*CKO LECs. Distinct receptor pathways upregulated in WT LECs involved the following: EPHA, CD226, ANGPT, OSM, NECTIN, and CD220 (Fig 5g).

We next quantified the similarity between all significant signaling pathways and then group them based on their cellular communication network using CellChat *NetSimilarity* function. Grouping was performed based on both functional and structural similarity of the lung cells expressing receptors. Application of the functional similarity grouping identified four groups of pathways both for WT and *Erg-*CKO (Fig 5h). For WT mice, Group #1,2 &3 were dominated by overlapping autocrine and paracrine signaling from myeloid, endothelial cells, and fibroblasts (e.g., Group #1- TGF-β, TNF, IL1, THY1, NOTCH, Group #2- COLLAGEN, PROS, GAS, Group #3- FGF, PDGF). Group #4 largely is dominant signaling from the endothelial cell (vascular and lymphatic) and mesothelial cell population- EPHB, VEGF, PECAM1, CDH5 (Fig. 5h, Supplementary Fig 5h). Group #1, which includes VEGF, SEMA3, NECTIN, and CDH5 pathways represents signaling from endothelial cells and inflammatory pathways In *Erg-*CKO mice. Group #2, which includes ANGPTL, ITGAL, RELN, and EDN1 represents signaling with high connectivity dominated by signals from endothelial cells, lymphatics, and myeloid cells. Group #3, which includes TGFb, THY1, PROS, and GAS pathways largely represents autocrine signaling between fibroblasts and myeloid cells. Group #4, which includes FGF, HGF, NOTCH, and TENASCIN pathways, represents autocrine signaling from endothelial cells and fibroblasts (Fig. 5i, Supplementary Fig 5i). Overall, CellChat predicts putative functions of the poorly studied pathways by grouping them together with pathways whose role is well known.

Structural similarity aggregation also identified four groups of signaling pathways (Fig 5j, k). For WT mice, Group #1 represented pathways with few senders and few receivers, such as TGFb, TNF, and VEGF. Group #2 represented pathways with numerous senders and few receivers, such as CDH1, and APRIL. Group #3 represented pathways with numerous receivers and few senders, such as BAFF, PERIOSTIN, and PTPRM. Group #4 represented pathways with numerous senders and few receivers, such as GAS, SEMA6, and CADM (Fig 5j, Supplementary Fig 5j). For *Erg-*CKO mice, Group #1 represented pathways with numerous receivers and few senders, such as L1CAM, TENASCIN, and WNT. Group #2 represented pathways with numerous receivers and few senders, EDN, IL1, and CD86. Group #3 represented pathways with very few senders and numerous receivers, such as VEGF, TNF, and NOTCH. Finally, Group #4 represented pathways with numerous senders and receivers, such as PARs, GAS, and OSM (Fig 5k, Supplementary Fig 5k). Overall, we identified key elements of intercellular communication within a given scRNA-seq dataset, which helps us predict the putative functions for poorly understood signaling pathways based on the size of the dataset.

### CellChat Identifies pulmonary LEC specific ligand-receptor communications impacted by *Erg* deletion.

**Figure 6:**
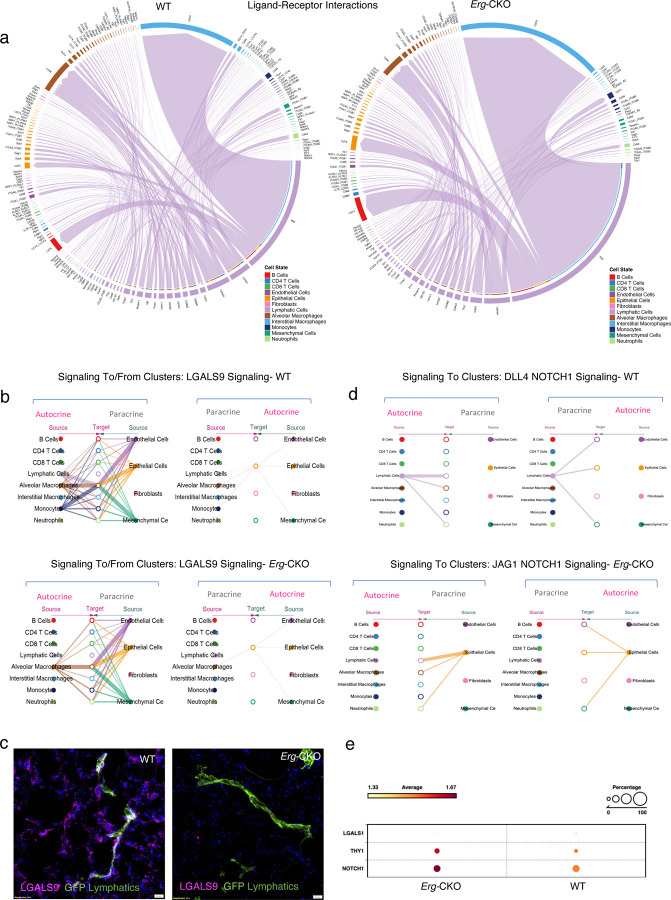
CellChat Identifies LEC specific ligand-receptor interacting pairs upregulated upon *Erg* deletion. **a,** CellChat netVisual chord gene analysis identifying significant ligand-receptor interactions amongst the single cell clusters in WT and *Erg-*CKO LECs represented as chord chart. **b,** CellChat hierarchy and network centrality plot indicating autocrine and paracrine directional interaction between LGALS9 and its receptors across single cell clusters in WT and *Erg-*CKO LECs. **c,** Colocalization immunofluorescence analysis of LGALS9 and GFP^+^ lymphatics in WT-GFP and *Erg-*CKO-GFP lung lymphatics (n = 3 WT vs 3 *Erg-*CKO). **d,** CellChat hierarchy and network centrality plot indicating autocrine and paracrine directional interaction between NOTCH1 and its receptors across single cell clusters in WT and *Erg-*CKO LECs. **e,** Differential gene expression highlighting changes in *NOTCH1, THY1, LGALS*.

We next utilized the CellChat *netVisual_chord_gene* and *netVisual_bubble* functionality to detect all the ligand-receptor interactions, more specifically, streamlining the communications to LEC-specific signaling. The CellChat *netVisual_chord_gene* analysis produced a LEC specific ligand-receptor interactions and pathway activation bubble and chord chart (Fig. 6a, Supplementary Fig 6a, b). CellChat identified loss of the following ligand-receptor communication from LECs to B cells in *Erg-*CKO lungs: LGALS9-CD45, LGALS9-IGHM, and MIF-(CD74+CXCR44). LECs to CD4 T-cells ligand-receptor communication found missing in *Erg-*CKO lungs were as follows: LGALS9-CD45, LGALS9-IGHM, and NECTIN2-CD26. More specifically, we found that APP, CDH5, THY1, VEGFA, VEGFD and SEMA6 ligand-receptor/pathway communications were elevated in *Erg-*CKO lungs (Supplementary Fig 6a, b). We also observed a reduction in LGALS9, COL4A1, PROS1, MIF, GRN, EPHA, and NOTCH1 ligand-receptor communication interactions in *Erg-*CKO LECs (Fig 6A, Supplementary Fig 6a, b). To further investigate these autocrine and paracrine ligand-receptor interactions, we used the CellChat hierarchy and network centrality plot to understand the directionality of interactions across cell types. CellChat identified that autocrine LGALS9 (GALECTIN9) signaling (LGALS9-CD45, LGALS9-IGHM, LGALS9-HAVCR2, and LGALS9-CD44) from LECs to other cell types is reduced in *Erg-*CKO lungs (Fig 6b, Supplementary Fig 7). LGALS9 is a key immune checkpoint protein that has been associated with the regulation of B cell trans-endothelial migration and cell-matrix interactions. LGALS9 overexpression has been speculated to be protective against fibroblast activation and humoral response in fibrotic lung diseases ^41,42^. Interestingly, IHC as well as single cell analysis confirmed a reduced LGALS9 localization in *Erg-*CKO lungs parenchyma and around the lymphatic vessels (Fig 6c, e).

We also observed changes in the NOTCH1 autocrine and paracrine signaling (signaling from: LECs to Alveolar macrophage, neutrophils) in WT *Erg-*CKO lung LECs. DLL4-NOTCH1 autocrine signaling between LECs, LEC-alveolar macrophage and LEC-neutrophil was robust in WT lungs whereas JAG1-NOTCH1 paracrine communication between epithelial cells and LECs was predominant in *Erg-*CKO LECs (Fig 6d). Compared to WT LECs, the expression of *NOTCH1* was significantly higher in Erg*-*CKO LECs (Fig 6E). Overall, CellChat’s hierarchy analysis provided key inputs regarding major signaling changes that may affect LECs’ pathophysiology.

### *Erg* deletion affects LEC transcriptomics In-Vitro.

**Figure 7:**
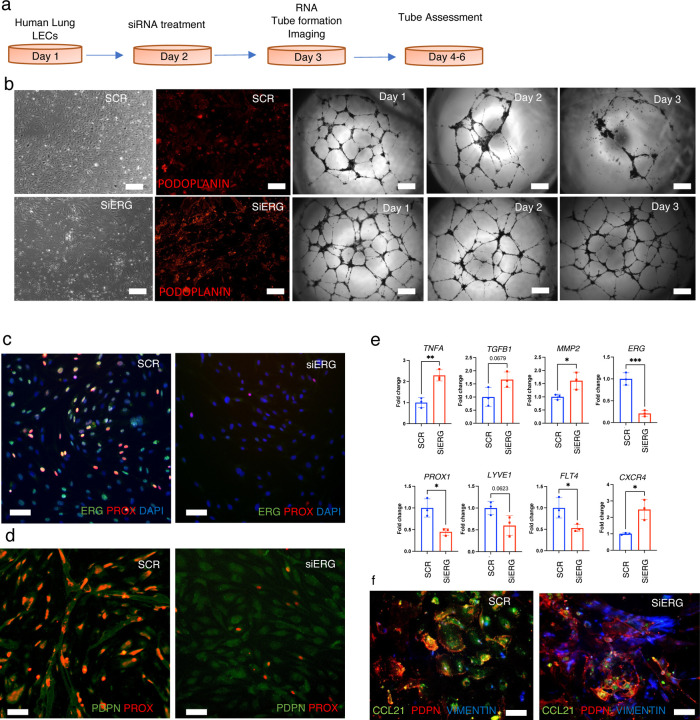
*Erg* deletion affects LEC transcriptomics In-Vitro. **a,** Diagrammatic outline of human lymphatic endothelial cell isolation protocol followed by in-vitro assessment of LEC genotype and phenotype. **b,** Assessment of tube formation phenotype for three days after siERG and siSCR treatment of the LECs. **c,** RNA expression of key lymphangiogenic and inflammatory genes in siERG and SCR treated LECs (n = 3 vs 3). **d,** ERG and PROX1 colocalization in 24 hr treated siERG and siSCR treated LECs (n = 3 vs 3.) **e,** PDPN and PROX1 colocalization in 24 hr treated siERG and siSCR treated LECs (n = 3 vs 3). **f,** CCL21(Green), PDPN (Red) and VIMENTIN (Blue) staining of LECs 48 hr after treatment with siERG and siSCR (n = 3 vs 3).

To investigate the effect of ERG deletion on lymphatic vessel remodeling, tube formation assays were performed. siSCR (scrambled negative control siRNA) and siERG treated LECs were monitored for tube network organization on Matrigel coated plates (see “methods“ section) (Fig 7a). siSCR LECs lost tube integrity after 72 h in culture; however, siERG LECs maintained robust tube networks. This corroborated with animal studies supporting lymphatic remodeling upon LEC specific ERG deletion (Fig 7b). Interestingly, siERG LECs continued expressing *PDPN*, suggesting no loss of lymphatic identity ^43^. We next examined the expression of pro-lymphangiogenic and pro-inflammatory genes in siSCR and siERG treated LECs. We observed a downregulation of *Flt4, Lyve1* and *Prox1* expression, meanwhile *Tgfb, Cxcr4, Mmp-2* and *Tnfa* expression increased in siERG LECs, supporting pro-inflammatory response by the LECs (Fig 7c). We also observe that the majority of the LECs with siERG treatment lose PROX1 and PDPN expression (Fig 7d, e). A majority of siERG treated LECs also transform to Vimentin expressing cells suggesting Endo-MT (Fig 7e). Overall, our data suggest that ERG expression is critical for lymphatic PROX1 expression and may be essential for maintaining lymphatic identity.

### Single-Cell Sequencing analysis identifies correlation between human IPF and *Erg-*CKO mice gene signatures.

**Figure 8:**
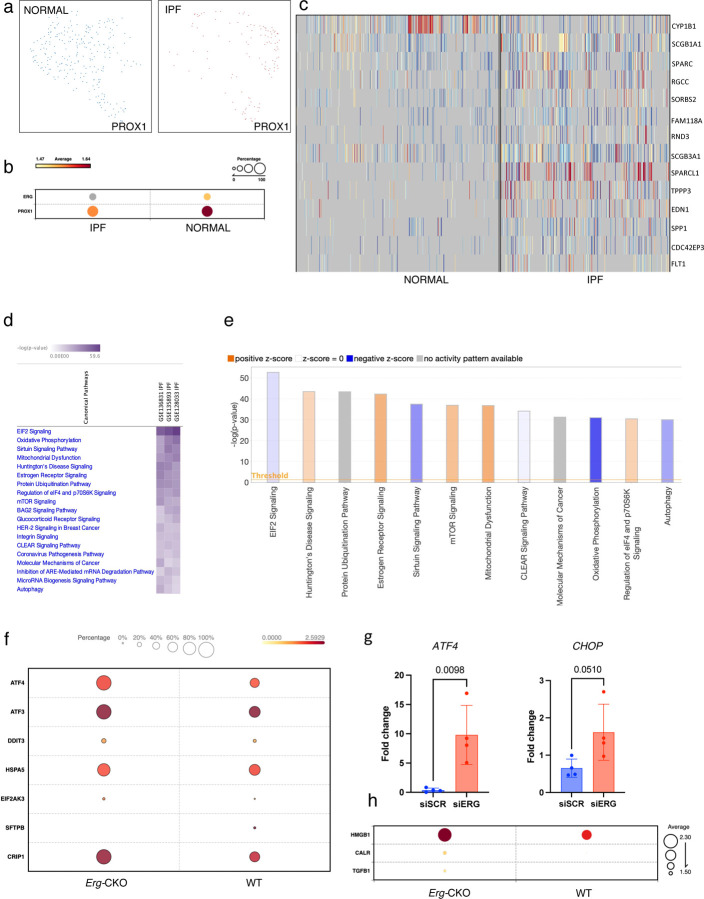
Single Cell Sequencing analysis identifies correlation between human IPF and *Erg-*CKO mice gene signatures. **a,** UMAP of healthy and IPF *Prox1* lymphatics clustered from GSE136831 IPF cell atlas using Biotouring-BBrowserX cell type prediction. **b,** Erg and Prox1 differential gene expression represented as bubble plot. **c,** Differential gene expression heatmap analysis identifying differences in gene signatures in healthy and IPF LECs. **d,** Ingenuity pathway prediction analysis suggesting activated pathways with Z score = 0–50 in IPF lungs. **e,** Ingenuity pathway prediction analysis highlights key pathways upregulated in and downregulated in *Erg-*CKO lungs (Z =0–50). **f,** Differential gene expression highlighting changes in expression of genes associated with ER stress and DNA damage repair as bubble-plots in *Erg-*CKO lungs. **g,** siERG treated human LECs upregulate *ATF4* and *CHOP* compared to siSCR LECs (n = 3 vs 3). **h,** Differential gene expression highlighting changes in gene expression associated with enhanced LEC immunogenicity in WT and *Erg-*CKO lungs.

We profiled three publicly available datasets of healthy and IPF lung (GSE128033, GSE136831, and GSE135893) yielding 3410 LECs using canonical lineage-defining marker *PROX1*. The LECs were UMAP clustered based on diseases state (healthy vs IPF) independent of the age and sex of the patients (Fig 8a, Supplementary Fig 8a, d). Both *ERG* and *PROX1* gene expression were downregulated in the three IPF cohorts (Fig 8b, Supplementary Fig 8b, e). Differential gene expression analysis identified several over-expressed genes in IPF LECs including *SCGBA1, SPARC, SFTPC, CRIP1, NTS, RAMP2, LDLRAD4, HSPG2, TBX1,* and *FLT1* (Fig 8c, Supplementary Fig 8c, f). Furthermore, Ingenuity pathway prediction analysis suggested activation of *EIF2* signaling (z score>50) based on collective upregulation of genes in IPF lungs, which has been linked to ER stress ^44^ (Fig 8d). Genes involved in mitochondrial dysfunction, oxidative phosphorylation, and autophagy were also upregulated in IPF LECs ^45–47^ (Fig 8d). Drawing parallels with IPF LECs, Ingenuity highlighted upregulation of EIF2, oxidative phosphorylation, and autophagy pathway associated genes in *Erg-*CKO LECs (WT vs *Erg-*CKO z<-1) (Fig 8e). More specifically, key genes associated with late onset of ER stress and DNA damage repair such as *Grp78 (Hspa5), Atf4, Atf3, Chop (Ddit3), Crip1*, and *caspase 12 (Eif2ak3)* were upregulated in the *Erg-*CKO collector LECs (Fig 8f). An increased expression of *Atf4* and *Chop* in siERG treated cultured human LECs further confirmed activation of ER stress response by LECs (Fig 8g). Upregulation of *Hmgb1, Calr, and Tgfβ1* also indicated an immunogenic phenotype of *Erg-*CKO LECs ^48–51^ (Fig 8h).

## Discussion:

In this study, we provide insights into the role of ERG in the regulation of lymphatic function and LEC transcriptomics. ERG expression is critical for blood endothelial cell (BEC) homeostasis; of note, mice with BEC-ERG deletion gain an accelerated interstitial fibrotic predisposition, similar to IPF lungs^14–16,52^. As IPF progresses, remodeling of the pulmonary interstitial vasculature encompasses an increase in blood capillary number and lymphatic vessel remodeling. Loss of subpleural and fibrotic scar lymphatics and dysfunctional lymphatic drainage further escalates fibrotic defects in lung tissue ^4,9^. A similar predilection to dysfunctional lymphatic drainage and LEC-mediated fibroblast activation has been made in a murine model of pulmonary fibrosis (IPF) ^53^.

We observe lymphatic vessel loss in the perivascular regions of IPF lungs. Majority of these human IPF lymphatic structures lacked nuclear ERG expression. Previous studies have shown that *Erg* expression is critical for vascular integrity, although, its suggested role in LEC junctional integrity is speculative. To determine the role of ERG in pulmonary lymphatics, *Erg-*CKO pulmonary lymphatic structures were characterized. Unlike acute exacerbated IPF lung tissues with dilated lymphatic vessels, we did not observe vessel dialysis in *Erg-*CKO mouse lung. However, *de novo* lymphatic remodeling in the alveolar spaces and an increase distribution of lymphatic vessels throughout the lungs was prominent, mimicking lymphatic remodeling pattens in early stages of IPF. Whole lung microarray and gene clustering analysis highlighted a collective upregulation of pro-inflammatory and fibrosis genes in the *Erg-*CKO lungs. High infiltration of LY6C+ monocytes ^30^, cDC1 antigen presentation cells^54^, and anti-inflammatory interstitial macrophages ^32^ collectively suggested an increased inflammatory predisposition in *Erg-*CKO lungs. Upregulation of key fibrotic genes (*Ccn2, Pai1*) and inflammatory genes (*Tgfβ, Ifnγr1* and *Ifnγr2)* in *Erg-*CKO lung further indicated a possible predisposition to fibrosis ^55–59^. Around 50% of our *Erg-*CKO mice also developed spontaneous tertiary lymphoid organ like B-cell structures. TLO formation is consequential for early inflammatory changes in IPF lung, whose persistent activation may trigger lymphatic mediated onset of fibrotic repair ^56^. Interestingly, *Erg-*CKO mice displayed no tissue fibrosis and formation of fibrotic foci. FITC-Dextran tracing analysis suggested an increased lymphatic vessel drainage in *Erg-*CKO mouse lung. The lack of pulmonary fibrosis was telling of the anti-fibrotic effects of increased interstitial lymphatic fluid drainage. Upregulation of key inflammatory mediators (such as TNF, *Tgf-β*1, Vegf, and Il-1β0029, known to enhance lymphatic permeability, pumping, and drainage, were also elevated in *Erg-*CKO lungs^60^. Inflammation concurs lymphangiogenesis ^61^ and lymphatic vessel remodeling is suggestive of active inflammation amelioration through lymphatic drainage modulation ^60–62^. Eventually, injury or persistent fibrosis of lungs may cause lymphatic insufficiency, a phenomenon which needs further validation in *Erg-*CKO lungs in an injury model.

Remodeling of pulmonary lymphatics is also associated with changes in vessel junction integrity ^39^. Downregulation of both tight junction and adherens junction genes in *Erg-*CKO pre-collector and collector lymphatics signified an impact on LEC junctional integrity. High *LYVE-1* expression in the collector LECs suggested an increase in metabolic degradation of hyaluronan and fluid uptake. Hyaluronan staining also confirmed localized accumulation along the collector and pre-collector lymphatic vessels and no distribution within the lung interstitium. Localized expression of hyaluronan along the lymphatic vessels confirmed non deterioration of lymphatic vessel integrity. The percentage of cells expressing tight junction genes were also significantly down in both pre-collector and collector *Erg-*CKO lymphatics. *Erg* deletion in pulmonary LECs, therefore, directly impacted the expression of genes associated with lymphatic junction integrity increasing lymphatic fluid flux as demonstrated through lymphatic drainage studies in lungs.

At a single-cell transcriptomic level, *Erg-*CKO LEC gene signatures drew parallels with IPF LECs. We observed that signaling pathways associated with EIF2, endoplasmic reticulum dysregulation, oxidative phosphorylation and stress were upregulated in *Erg-*CKO pulmonary LECs. Although we did not observe a consistency in the pathway activation z-scores, genes associated with stress had a high p-value score indicating that LECs can functionally regulate stress genes to detect physiological changes in lung tissue ^63^. Stress and inflammation induced lymphatic remodeling has been previously demonstrated in numerous studies ^64,65^. Key indicators of oxidative and ER-stress associated genes, such as SPARC, SCGB1A1 and ATF4, were upregulated in IPF LECs^66,67^. The expression pattern of SPARC, SCGB1A1, HMGB1, CALR, TGF-B and ATF4 in *Erg-*CKO LECs suggest a response aligning to IPF ER and oxidative stress. We are yet to validate if these ER and oxidative stress factors are the primary indicators of an increased LEC function.

Our multi-omics gene analysis clustered with CellChatBD R-package further highlighted key LEC specific cell-cell communication/ ligand-receptor interaction impacting LEC function ^68^. Ligands involved with LEC mediated fibroblast activation, Endo-MT, endothelial and inflammatory cell signaling were identified ^42,69–75^. These *Erg-*CKO communication ligands were THY1, VEGFA, LGALS9, NOTCH1, and SEMA6A. *NetSimilarity* and *NetVisual chord gene* analysis further streamlined ligand-receptor communication networks that were downregulated (such as: COL4A1, PROS1, MIF, GRN, EPHA, and NOTCH1) and upregulated (APP, CDH5, and THY1) in *Erg-*CKO LECs.

THY1 which is expressed in LECs and has been shown to support memory-pathogenic T helper 2 cell maintenance ^76,77^, and has been shown to mitigate pulmonary fibrosis ^75^. We observe an increase in LEC-myeloid cell interaction though THY1-(ITGAM/ITGB2), THY1-(ITGAX/ITGB2) autocrine and paracrine signaling, which is supportive of an inflammatory immune cell predisposition in lungs^78,79^. We also observe upregulation of NOTCH1 and downregulation of LGALS9 expression in *Erg-*CKO LECs. Studies show that NOTCH1 signaling is critical for postnatal lymphatic development and pathogenic lymphangiogenesis ^80,81^. NOTCH1 is also a critical regulator of pro-fibrotic alveolar epithelial cells proliferation and onset of endothelial cell myofibroblast transition (Endo-MT) in IPF lungs ^82,83^. Our CellChatBD ligand-receptor directional analysis indicates pulmonary LEC specific autocrine JAGGED1-NOTCH1 signaling to epithelial cells in *Erg-*CKO LECs. Sheer stress induced Vimentin phosphorylation and Jagged1-Notch1 transactivation has been shown in endothelial remodeling and Endo-MT ^84,85^. ERG expression may therefore directly or indirectly regulate NOTCH1 expression in LECs. LGALS9 is another emerging immune checkpoint inhibitor ^86^ ligand whose autocrine communication with CD45, IGHM, HAVCR2 and CD44 as inhibited in Erg-CKO LECs. LGALS9 is a prognostic marker of pulmonary fibrosis, collagen vascular diseases, ILD, although its function in IPF lung remains unknown ^41,42,87–89^. Furthermore, there are no studies linking lymphatic function and LGALS9. A reduced ecto- LGALS9 in *Erg-*CKO lung parenchyma and around LECs corroborated with the gene expression levels. A further validation of these targets would provide insight in pulmonary lymphatic functions.

Finally, our in-vitro analysis revealed that loss of lymphatic ERG downregulated the expression of *PROX1* in hLECs. *PROX1* is the master regulator of LEC cell identity ^90^. Our invitro analysis, in corroboration multi-omics results which demonstrate *ERG* and *PROX1* downregulation in IPF lung indicate the direct role of Erg in regulating lymphatic identity genes. In-vitro, other key lymphatic identity gene downregulated in siERG treated LECs was Flt4. siERG treated LECs also upregulated pro-inflammatory genes (*TNFA, TGFB*) and ER-stress genes (*CHOP, ATF4*). The hLECs also start expressing vimentin suggesting their Endo-MT transformation. Interestingly these siERG treated LECs maintained a robust tube network without the loss of PDPN, confirming a direct role in vessel remodeling.

In conclusion, our results provide insights on the role of vascular endothelial identity gene, *Erg,* in lymphatic function. Lymphatic remodeling in *Erg-*CKO lungs seem to be protective against fibrosis, although it would be interesting to observe pulmonary predisposition in *Erg-*CKO lungs during stress and injury. Identifying LEC specific communication pathways and ligand-receptor targets will then be critical to develop strategies aimed to reducing pulmonary fibrosis through lymphatic remodeling in the lungs.

## Methods:

### Human tissues and cell cultures.

Healthy human lung biopsies were obtained from three healthy and four IPF donors in compliance with the Institutional Review Board (IRB) for Human Studies. LECs used in in vitro studies were obtained from adult healthy individuals undergoing abdominoplasty. The lung sections were incubated with collagenase II (1mg/ml) (Thermofisher) and dispase II (1mg/ml) (Thermofisher) /DMEM for two hours in 37 ℃ water bath. Debris was removed by sequential filtration through 70 μm filters (BD Biosciences, San Jose, CA) and cell mixture was cultured for few days in endothelial cell basal medium MV2 with supplements (PromoCell, Heidelberg, Germany). Endothelial cells were purified from cell mixture with CD31 positive magnetic beads (Dynabeads) (Thermofisher) and cultured until beads were detached from the cells. LECs were purified from endothelial cells with *PDPN* positive magnetic beads (anti-PDPN rat antibody, anti-Rat sheep magnetic beads) and cultured in endothelial cell basal medium MV2 with supplements. 24 hours before harvest medium was changed with an addition of VEGFC (100ng/ml) (Sino Biological).

### Mice.

All animal experimental procedures were performed in accordance with Protocol 201800144_TR01 reviewed and approved by the Boston University Institutional Animal Care and Use Committee. The investigation conformed to the Guide for the Care and Use of Laboratory Animals, published by the US National Institutes of Health (NIH).

Generation of *Erg* floxed mice was previously described ^20^. *Erg*^fl/fl^ mice were purchased from Jackson laboratory. *Prox1*-CreER(T) were purchased from Taconic. Conditional *Prox1*-CreER(T)-*Erg*^*fl/fl*^ on a C57/BL6 background were generated by breeding of *Prox1*-CreER(T)-mice with *Erg*^*fl/fl*^ mice. We also generated *Erg-CKO-*GFP mice by breeding *Prox1*-CreER(T)-tdTomato mice with *Erg*^*fl/fl*^-GFP mice. Mice had access to food and water ad libitum and were on a 12h/12h light/dark cycle. 100ul of 20mg/ml tamoxifen injection are injected for 5 days to induce *Erg* deletion. 30 days after tamoxifen injections both WT and *Erg*-CKO mice used for experiments.

### Immunohistochemical analysis.

Human or mouse skin tissues were fixed with 4% paraformaldehyde in PBS. 5-μm sections after dewaxing and heat antigen retrieval in Tris/EDTA pH 9.0 for 20 minutes were used for staining. Blocking was achieved using 3% H_2_O_2_ followed by BLOXALL (Vector Labs, Burlingame, CA) or 2% horse or goat serum. Appropriate Vector HRP-ImmPress Polymers (mouse, rat, and rabbit) were used to detect primary antibodies. Immunohistochemistry was also performed on formalin-fixed, paraffin-embedded tissue sections using a Vectastain ABC kit (Vector Laboratories, Burlingame, CA) according to the manufacturer’s instructions. The concentration of primary antibody was first tested to determine the optimal sensitivity range. Antibodies have been listed in Table 1.

### RNA extraction, quantitative RT-PCR.

RNA was isolated from LECs using the TRI Reagent (MRC Inc.). 1 μg of total RNA was reverse transcribed with random hexamers using the Transcriptor First Strand cDNA Synthesis kit (Roche Applied Science) according to the manufacturer’s protocol. Diluted cDNA was mixed with SYBR^®^ Green PCR Master Mix (Applied Biosystems) to quantitatively measure gene expression using a StepOnePlus Real-Time PCR system (Applied Biosystems). Relative change in the levels of genes of interest was determined by the 2-ΔΔCT method using housekeeping genes. Expression of the housekeeping genes GADPH served as internal references in each assay performed. The list of primers tested have been mentioned in Table 2.

### siRNA transient transfection.

70% confluent cultures of LEC were transfected with 10 nM of small interfering RNA (siRNA) directed against ERG (ON-TARGET plus SMART pool^™^, Dharmacon, Waltham, MA) and control siRNA using Invitrogen^™^ Lipofectamine^™^ RNAiMAX Transfection Reagent (Invitrogen). 24 hours post-transfection, total RNA was prepared using TRI reagent (MRC, Inc., Cincinnati, OH) according to the manufacturer’s protocol. 48 hours post-transfection, whole cell lysates were prepared for immunoblot analysis, or functional assays were performed.

### Matrigel assay.

Tube formation assays were performed by applying LEC treated with siRNA for 48h before seeding in Matrigel (20,000 cells/96well, Corning; Glendale, AZ), followed, where indicated, by incubation for 18 hours with adeno virus expressing FLT4 or control virus. The number of tubes in each well were counted under microscope.

### Flow Cytometry.

Mice were anesthetized with ketamine/xylazine solution (100 and 10 mg/kg, respectively), injected intraperitoneally, and perfused via the left ventricle with cold PBS 30 days after bleomycin or PBS delivery. The lungs were immediately harvested and minced with a razor blade in a 100 mm petri dish in a cold DMEM medium containing 0.2 mg/ml Liberase DL and 100 U/ml DNase I (Roche, Indianapolis, IN, USA). The mixture was transferred into 15 ml tubes and incubated at 37 °C for 35 min in a water bath under continuous rotation to allow enzymatic digestion. Digestion was inactivated with a DMEM medium containing 10% fetal bovine serum, the cell suspension was passed through a 40 µm cell strainer (Fisher, Waltham, MA, USA) to remove debris. Cells were then centrifuged (500×*g*, 10 min, 4 °C), and resuspended in 3 ml red blood cell lysis buffer (Biolegend, San Diego, CA, USA) for 90 s to remove the remaining red blood cells and diluted in 9 mL PBS after incubation. Cells were then centrifuged (500×*g*, 10 min, 4 °C) and resuspended in 0.2 ml of FACS buffer (1% BSA, 0.5 mM EDTA pH 7.4 in PBS). For analysis, the single-cell suspension derived from lungs of WT and ERG CKO mice was obtained as described above and then incubated with antibodies listed in Table 3. The immune cells were then characterized based on specific membrane markers as following: Neutrophils (CD45^+^, CD11b^+^, Ly6G^+^), NK cells (CD45^+^, NK1.1^+^, Ly6G^+^), Monocytes (CD45^+^, CD11b^+^, Ly6C^+^), Macrophages (CD45^+^, SiglecF^+^, F4/80^+^), Eosinophils (CD45^+^, CD11b^+^, SiglecF^+^), B cells (CD45^+^, CD19^+^, B220^+^), CD4^+^ T cells (CD45^+^, CD4^+^), CD8^+^ T cells (CD45^+^, CD8^+^). FACS analysis was conducted using a Cytek Aurora 5L (Cytek Biosciences, Fremont, CA, USA). Data were analyzed with FlowJo version 10.8.0 software (Tree Star Inc., Ashland, OR,SA).

### Microarray.

All procedures were performed at Boston University Microarray Resource Facility as described in the GeneChip^®^ Whole Transcript (WT) Plus Reagent Kit Manual (Affymetrix, Santa Clara, CA). Briefly, the total RNA was isolated using RNeasy kit (Qiagen), and the sample integrity was verified using RNA 6000 Pico Assay RNA chips run in Agilent 2100 Bioanalyzer (Agilent Technologies). The total RNA form WT and *Erg*-CKO lungs (100 ng) was reverse transcribed using GeneChip^®^ WT PLUS Reagent Kit (Affymetrix). The obtained cDNA was used as a template for in vitro transcription using GeneChip^®^ WT Expression Kit (Life Technologies). Antisense cRNA was then purified using Nucleic Acid Binding Beads (GeneChip^®^ WT PLUS Reagent Kit, Affymetrix) and used as a template for reverse transcription to produce single-stranded DNA in the sense orientation. During this step, dUTP was incorporated. The DNA was then fragmented using uracil DNA glycosylase (UDG) and apurinic/apyrimidinic endonuclease 1 (APE 1), followed by labeling with DNA labeling reagent covalently linked to biotin using terminal deoxynucleotidyl transferase (TdT, GeneChip^®^ WT PLUS Reagent Kit, Affymetrix). IVT and cDNA fragmentation quality controls were carried out by running an mRNA Pico assay in the Agilent 2100 Bioanalyzer. The labeled fragmented DNA was hybridized to the Gene Arrays 1.0ST for 16–18 h in GeneChip^®^ Hybridization oven 640 at 45 °C with rotation (60 r.p.m.). The hybridized samples were washed and stained using Affymetrix fluidics station 450 as per manufacturer’s instruction (Hybridization, Washing and Staining kit, Affymetrix). Microarrays were immediately scanned using Affymetrix GeneArray Scanner 3000 7G Plus (Affymetrix).

### 10X 3’v3 Single Cell Gene Expression Library Preparation & Sequencing.

Cell viability and counts were determined using hemocytometer and trypan blue. Single cell sequencing libraries were generated according to 10X Genomics’ Chromium Single Cell 3ʹ GEM, Library & Gel Bead Kit according to the manufacturer’s protocol (10X Genomics, USA). Briefly, Single Cell 3’ Gel Beads containing unique bead barcodes and transcript UMIs were combined with single cells in suspension and partitioning oil, before loading onto a Chromium Chip G to generate GEMs (Gel Bead in Emulsion). To achieve single cell resolution, cells are delivered at a limiting dilution, such that the majority (90%-99%) of generated GEMs contain one Gel Bead and no cell, while the remainder largely contain both a Gel Bead and a single cell. 10,000 cells with a range of 60–83% viability was targeted for capture and library preparation. After GEMs generation, the Gel Bead was dissolved, followed by incubation to produce barcoded, full-length cDNA from poly-adenylated mRNA. GEMs were then broken, and pooled fractions were amplified via PCR (16 cycles) to generate sufficient mass for library construction. A Bioanalyzer High Sensitivity DNA Assay (Agilent Technologies, USA) was used to determine size distribution and yield of amplified cDNA. Double-stranded cDNA then underwent fragmentation, end repair, A-tailing, and adaptor ligation. Incorporation of sample-specific multiplex indices occurred during PCR amplification (14–16 cycles) according to the manufacturer’s protocol (10X Genomics, USA). Size distribution and molarity of amplified cDNA libraries were assessed via the Bioanalyzer High Sensitivity DNA Assay (Agilent Technologies, USA). All cDNA libraries were sequenced on an Illumina NextSeq 2000 instrument (Illumina, USA) targeting 50 thousand reads per cell per sample. The following subclassifications were made to identify the lung cell subtypes: Alveolar Macrophages (*Ear2*), NK cells (*Gzma*), B cells (*Cd79b*), Plasma cells (*Mzb1*), CD4+ T cells (*Trbc2*), CD8+ T cells (*Trbc2, Cd8b1, Ly6c2*), Non-classical monocyte (*Ly6c2-,Plac8*), Interstitial macrophages (*C1qb*), Classical monocyte (*Ly6c2+, Plac8*), CD103+/CD11b-_DCs (*Itgae*), Ccl17+/CD103-/CD11b-_DCs (*Ccl17*), CD209+/CD11b+_DCs (*Ccl17, Cd209a*), Tregs (*Cd3+, Cd4+, Foxp3+*), Neutrophils (*Ngp, Ly6c2*), Eosinophils (*Cxcr2*), Endothelial cells (*Cdh5, Pecam1*), Lymphatic endothelial cells (*Prox1*) and Alveolar Fibroblasts (*Pdgfra, Slc7a10*), Adventitial Fibroblasts (*Pi16, Dcn*), Peribronchial Fibroblasts (*Fgf18, Hhip*), and Activated Fibroblasts (*Col1a1, Fn1*)

### Clustering of single-cell data with Celda and Biotouring BbrowserX.

The *celda* package, as well as biotouring package, was used to bi-clustering genes from WT and Erg-CKO lungs into modules and cells into subpopulations^3^. Features with less than 3 counts in 3 cells were excluded, then the 5,000 most variable features were identified using the *seuratFindHVG* function from the *singleCellTK* package. The *recursiveSplitModule* and *recursiveSplitCell* functions were used to select the model with 50 gene modules and 15 cell subpopulations after examining the Rate of Perplexity Change (RPC). Cells were embedded in two dimensions with UMAP using the *celdaUmap* function. Heatmaps for specific modules were generated using the *moduleHeatmap* function. Markers for each cluster were identified with the *findMarkerDiffExp* function from the *singleCellTK* package using the Wilcoxcon test and an FDR threshold of 0.05. The cell type identities were then assigned to clusters by investigating the expression of canonical markers. WT and ERG CKO feature, and barcode matrices were also imported into BioTuring Browser 2 (Bioturing, San Diego, USA). A total of 25888 cell profiles were obtained after quality filtering from WT mice and ERG-CKO mice. A t-Distributed Stochastic Neighbor Embedding (t-SNE) dimensionality reduction method combined with canonical correlation analysis (CCA) subspace alignment is used to reduce dimensionality. Unsupervised graph clustering is then performed. Human IPF single-cell RNA-seq data23 were obtained from the BioTuring repository and analyzed with the BioTuring Browser 2.

### Inference of cell-cell communication with CellChat.

The *CellChat* package was used to infer and visualize the cell-cell communications, with the log-normalized counts and cell identities as the inputs. The communication probabilities were computed by identifying over-expressed genes and interactions and using the *computeCommunProb* function with default settings and filtering communications with less than 10 cells, then pathway probabilities were computed using the *computeCommunProbPathway* function. Pathways with significant interactions were visualized with the *netVisual* function. Signaling roles of each cell type were identified using the *netAnalysis_computeCentrality* function. Outgoing and incoming communication patterns were identified with the *identifyCommuncationPatterns* function, selecting the number of patterns using the *selectK* function and identifying the value where the stability scores begin to drop suddenly. Signaling pathways were grouped based on their functional and structural similarities using the *computeNetSimilarity* function and embedded in two dimensions with UMAP using the *netEmbedding* function then clustered with k-means using the *netClustering* function.

### Statistics.

Analyses were performed using Prism 9 (GraphPad) software. Values are presented as means ± SD. Differences between more than two groups were compared with ANOVA with a post hoc Tukey test or Kruskal–Wallis tests followed by post hoc pairwise Dunn multiple comparison tests. Two-sided student t test or Mann Whitney U test were used for comparing 2 groups. Correlations are Spearman analyses with Holm–Sidak adjusted p values. In all cases, p<0.05 were considered significant and are abbreviated in the figures as follows: *p < 0.05, **p < 0.01.

## Supplementary Material

Supplement 1

## Figures and Tables

**Table 1:** Antibody List

Staining Antibody	Cat no.	Company	RRID
DAPI	H-1200	Vector Laboratories	AB_2336790
PDPN	14-9381-82	Thermo Fisher Scientific	AB_1603307
ERG	97249	Cell Signaling Technology	AB_2721841
PROX1	ab199359	Abcam	AB_2868427
KI67	ab15580	Abcam	AB_443209
PTX3	ITT3905-100u-647	G-Biosciences	
LYVE1	AF2125	R&D Systems	AB_2297188
EGFR	NB600-724	Novus	AB_10002344
CCL21	MAB457	R&D Systems	AB_2259799
VIMENTIN	ab137321	Abcam	AB_2921312
GALECTIN9	ab69630	Abcam	AB_1268942

**Table 2:** Primer List

Primer	Species	Forward	Reverse
*GAPDH*	H	GGTCTCCTCTGACTTCAACA	AGCCAAATTCGTTGTCATAC
*TNFA*	H	GAGGCCAAGCCCTGGTATG	CGGGCCGATTGATCTCAGC
*TGFβ1*	H	GCAGCACGTGGAGCTGTA	CAGCCGGTTGCTGAGGTA
*MMP2*	H	CCCCAAAACGGACAAAGAG	CTTCAGCACAAACAGGTTGC
*ERG*	H	CCAGTCGAAAGCTGCTCAA	GTTGGTCCAAGAATCTGATAAGG
*PROX1*	H	AAATATCACCTTATTCGGGAAGTG	TTTTCAAGTGATTGGGTGACAA
*LYVE1*	H	AGGCTCTTTGCGTGCAGAA	GGTTCGCCTTTTTGCTCACAA
*FLT4*	H	TGCACGAGGTACATGCCAAC	GCTGCTCAAAGTCTCTCACGAA
*CXCR4*	H	ACTACACCGAGGAAATGGGCT	CCCACAATGCCAGTTAAGAAGA
*ATF4*	H	TTCTCCAGCGACAAGGCTAAGG	CTCCAACATCCAATCTGTCCCG
*CHOP*	H	GGAAACAGAGTGGTCATTCCC	CTGCTTGAGCCGTTCATTCTC
			
*Gapdh*	M	TGCCCCCATGTTTGTGATG	TGTGGTCATGAGCCCTTCC
*Pai1*	M	AGGATCGAGGTAAACGAGAGC	GCGGGCTGAGATGACAAA
*Mmp12*	M	TGATGCTGTCACAACAGTGG	GTAATGTTGGTGGCTGGACTC
*Ifng2*	M	TCCTGTCACGAAACAACAGC	ACGAATCAGGATGACTTGC
*Ccn2*	M	GGGCCTCTTCTGCGATTTC	ATCCAGGCAAGTGCATTGGTA
*Pdgfb*	M	TTGCAACGAGAAAGCCGGA	CTATCTACCCACTCGCTCGC
*Tgfb1*	M	CTCCCGTGGCTTCTAGTGC	GCCTTAGTTTGGACAGGATCTG
*Vegfa*	M	GGCCTCCGAAACCATGAACTT	TGGGACCACTTGGCATGGTG
*Vegfc*	M	CGTTCTCTGCCAGCAACATTACCAC	CTTGTTGGGTCCACAGACATCATGG
*Vegfd*	M	GCAACTTTCTATGACACTGAAACAC	TCTCTCTAGGGCTGCATTGG

**Table 3:** Flow Cytometry Antibody List

FLOW Antibody	Cat no.	Company	RRID
PerCP Cy5.5 CD45	45-0451-80	Thermo Fisher Scientific	AB_906233
PE-CY7 CD11c	558079	BD Biosciences	AB_647251
APC-CY7 SIGLEC F	565527	BD Biosciences	AB_2732831
BV421 CD11b	101235	BioLegend	AB_10897942
APC CD4	100411	BioLegend	AB_312696
PE-Fire640 CD8a	100789	BioLegend	AB_2860590
BUV395 CD19	563557	BD Biosciences	AB_2722495
BV711 MHCII	107643	BioLegend	AB_2565976
PE-DAZZLE594 CD103	121429	BioLegend	AB_2566492
BV510 CD24	101831	BioLegend	AB_2563894
PE CD64	139304	BioLegend	AB_10612740
FITC-Ly6G	127606	BioLegend	AB_1236494
Bv605 Ly6C	563011	BD Biosciences	AB_2737949
eFluor Ly6C	48-5932-80	Thermo Fisher Scientific	AB_10805518
