# Supplementary material for: Endothelial Erg Regulates Expression of Pulmonary Lymphatic Junctional and Inflammation Genes in Mouse Lungs Impacting Lymphatic Transport
Source: Res Sq. 2024 Jan 24:rs.3.rs-3808970. Preprint. [Version 1] doi: 10.21203/rs.3.rs-3808970/v1 (PMC10854286; doi:10.21203/rs.3.rs-3808970/v1)
Supplement: Supplement 1 [file NIHPPrs3808970v1-supplement-1.pdf]

Supplementary Files

This is a list of supplementary files associated with this preprint. Click to download.

- SupplementaryFiguresAll.pdf
